# LIFESTYLE TRAJECTORIES PREDICT LATER COGNITIVE FUNCTION AMONG OLDER PEOPLE IN TAIWAN

**DOI:** 10.1093/geroni/igad104.3107

**Published:** 2023-12-21

**Authors:** Hui-chuan Hsu

**Affiliations:** Taipei Medical University, New Taipei, New Taipei, Taiwan (Republic of China)

## Abstract

**Purpose:**

The purpose of this study was to identify the group-based trajectories of lifestyles and to predict the later cognitive function among older people in Taiwan.

**Methods:**

The data were from five waves of the “Taiwan Survey on Aging “ (1999-2015). Only those who completed the last wave of cognitive function were used for analysis (n=1314). The cognitive function was measured by 8 items of SPMSQ, reverse numbers and recall 10 words, with score ranged 0-19. Nineteen lifestyle behaviors (healthy behaviors, leisure activities, lifelong learning and social participation) across waves were analysed by group-based trajectory analysis first, and then the effects of lifestyle trajectories on cognitive function were analysed by linear regression.

**Results:**

Each lifestyle variable was identified as 2 to 6 trajectory groups. Significant lifestyle trajectories on later cognitive function included gardening (high), drinking (increasing), providing instrumental support (increasing), playing chess (high), reading (middle & high), outdoor activities (high), group activities (yes), political participation (no), volunteering (yes), and stress (high vs. lowest). After controlling for covariates, cognitive function was related to gardening (high) (B=0.642), playing chess (high) (B=0.855), reading (high) (B=1.699), group activities (B=0.640), and lower stress (high stress B=−0.806). Other significant variables for better cognitive function included younger age, living with less family members, having less physical function difficulties, having lower depressive symptoms, and being more religious.

**Conclusion:**

Long-term lifestyle trajectories may affect later cognitive function. Having more social participation, more cognitive-related leisure, having less stress and being physically healthier are protective for cognitive function.

